# Supplementary material for: An Inducible Luminescent System to Explore Parkinson’s Disease-Associated Genes
Source: Int J Mol Sci. 2024 Aug 31;25(17):9493. doi: 10.3390/ijms25179493 (PMC11395715; doi:10.3390/ijms25179493)
Supplement: Supplementary file 1 [file ijms-25-09493-s001.zip › ijms-3107164-supplementary.pdf]

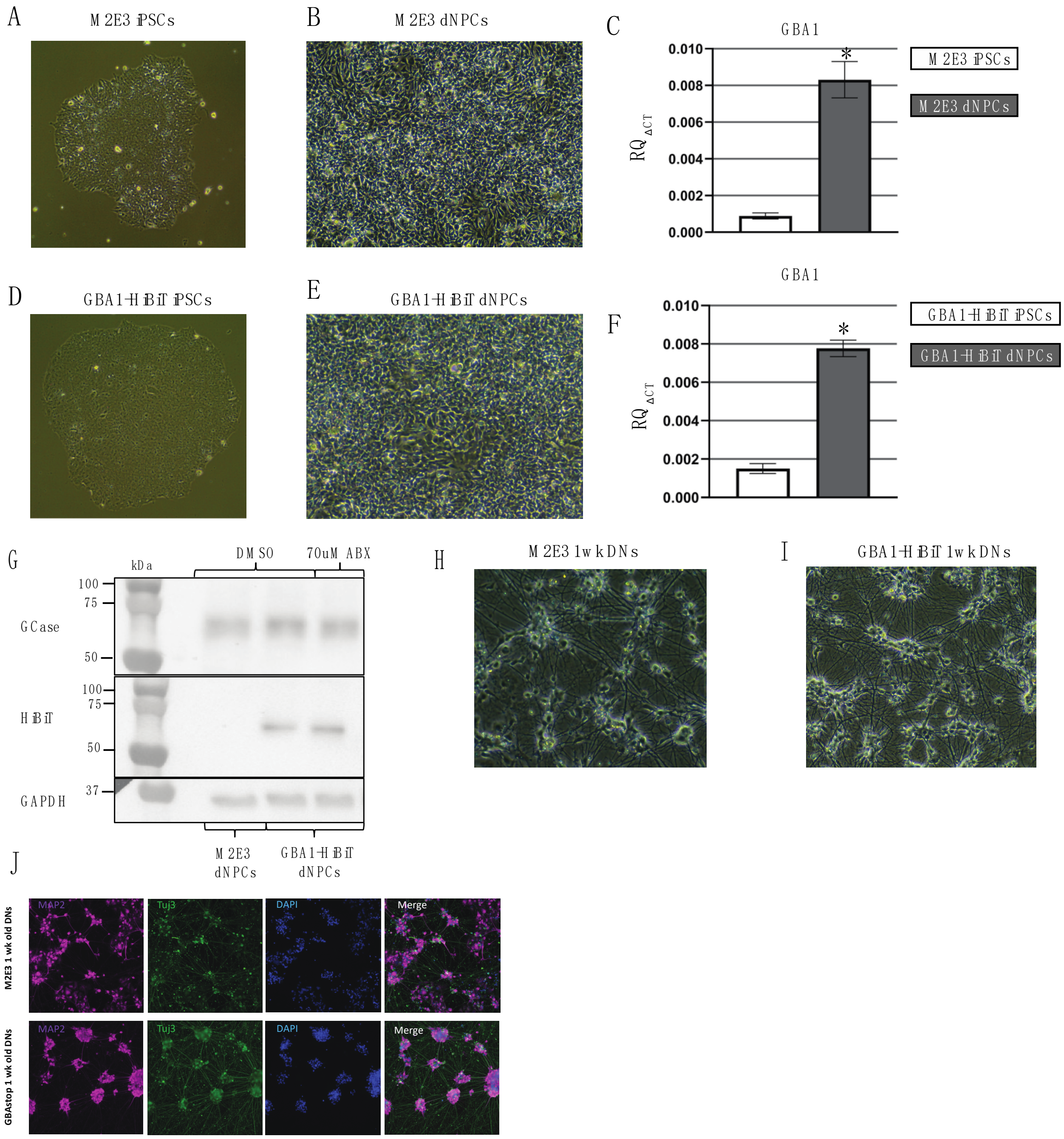

Figure S1. Quality control experiments for M2E3 and GBA1-HiBiT cells and validation of LgBiT-HiBiT system in 1-week-old DNs. A) Brightfield images of M2E3 iPSCs and B) dNPCs. C) Transcript levels evaluated by RT-qPCR of *GBA1* in M2E3 iPSCs and dNPCs. Values are presented as mean ± SD of three experiments, each including three technical replicates. Two-tailed paired t-test was used to compare iPSC and dNPC samples; $*p < 0.0001$. D) Brightfield images of GBA1-HiBiT iPSCs and E) dNPCs. F) Transcript levels evaluated by RT-qPCR of *GBA1* in GBA1-HiBiT iPSCs and dNPCs. Values are presented as mean ± SD of three experiments, each including three technical replicates. Two-tailed paired t-test was used to compare iPSC and dNPC samples; $*p<0.0001$. G) Immunoblotting of HiBiT, GCase, and GAPDH in M2E3 and GBA1-HiBiT dNPCs, treated or not with 70 μM Ambroxol for 6 days. H) Brightfield images of M2E3 and I) GBA1-HiBiT 1-week-old DNs. J) ICC staining of MAP2, Tuj3, and DAPI in 1-week-old GBA1-HiBiT DNs.

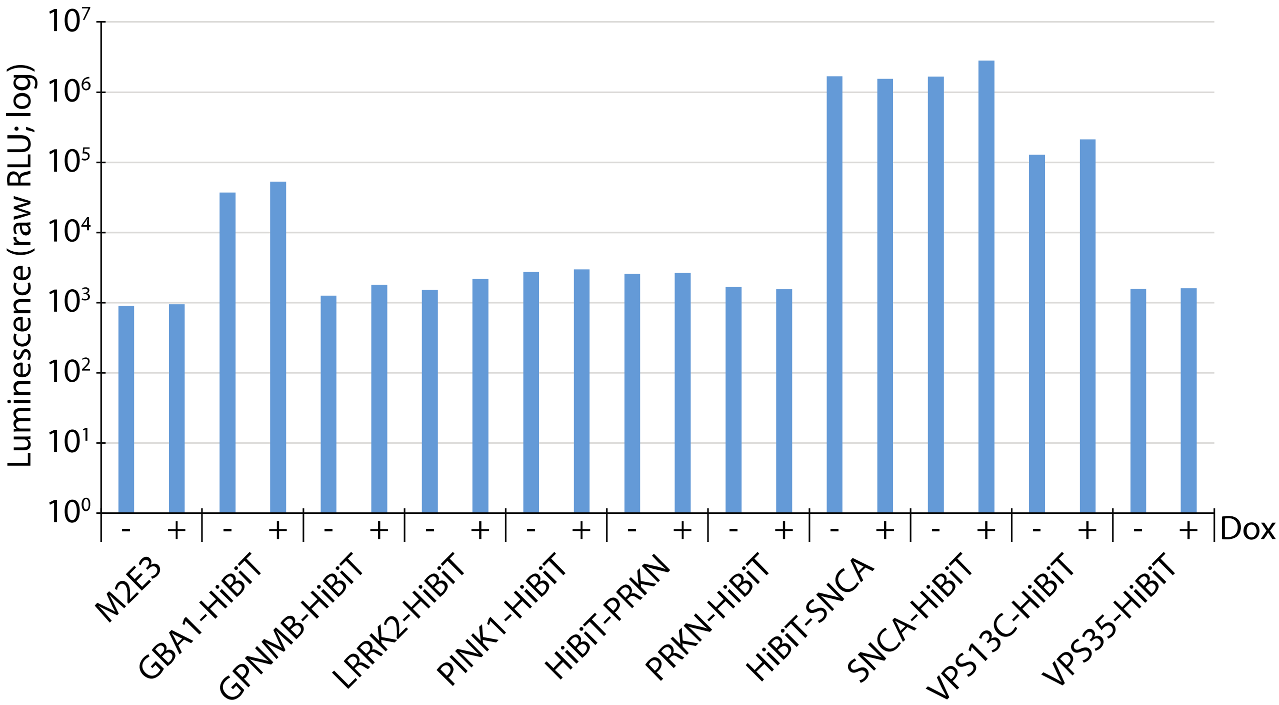

Figure S2. Bar graph showing luminescence assay readout in master M2E3 iPSC line, as well as in the different HiBiT knock-in iPSC lines treated (+) or not (-) with Dox. Raw relative light unit (RLU) values from a single experiment are presented, including one technical replicate for each line. Note that this data set was extracted from the same experiment presented in Figure 3E.

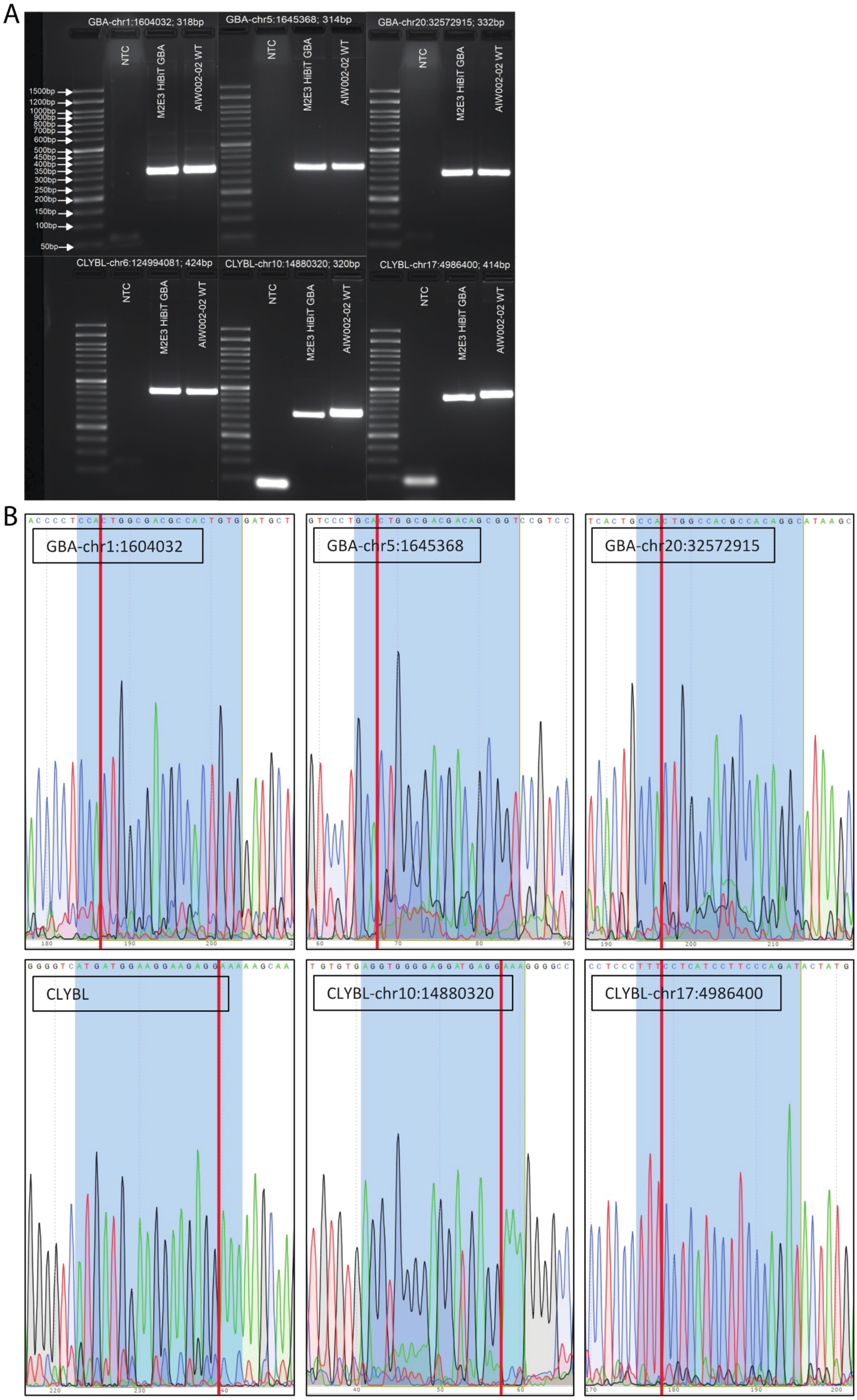

Figure S3. Off-target indel analysis of the top 3 potential sites, as ranked by Benchling, for both sgRNA-GBA1 and sgRNA-CLYBL. A) PCR amplification of

the selected off-target regions in both edited iPSC lines and in control AIW002-02 iPSCs. B) Sanger sequencing chromatogram corresponding to each extracted PCR band from edited lines in A). The chromosomal location is labeled at the top left of each output, with the shaded blue area corresponding to the sequence that resembles that of the used sgRNA with a few mismatches. The red line denotes the predicted cutting site in the event of off-target activity.
